# A geminivirus attenuation vector for crop protection using episomal plant gene therapy

**DOI:** 10.1038/s41598-025-09038-3

**Published:** 2025-07-11

**Authors:** Natalie Thompson, Rekha Kandaswamy, Aliya Fathima Anwar, Jane Polston, Garry Sunter, Wayne R. Curtis

**Affiliations:** 1 Department of Chemical Engineering, The Pennsylvania State University, University Park, PA 16802 USA; 2 Department of Plant Pathology, University of Florida, Gainesville, FL 32611 USA; 3 Department of Biology, University of Texas, San Antonio, TX 78249 USA; 4 Intercollege Program in Plant Biology, The Pennsylvania State University, University Park, PA 16802 USA

**Keywords:** Geminivirus, Biocontainment, Antiviral treatment, Bioluminescence, Plant biotechnology, Biotechnology, Gene therapy, Chemical engineering

## Abstract

Although plant viruses inflict billions of dollars in global crop yield loss, they also encode functions that can be used for crop protection. A viral vector attenuation strategy is developed that enables modulation of a ‘gene therapy’ viral vector designed for protective gene delivery. This attenuation vector targets its homologous native viral counterpart, where the A-component of the bipartite tomato mottle virus (ToMoV) viral vector delivers siRNA constructs targeting native viral sequences for which its own genome has been synthetically modified to avoid silencing. A target coat protein replacement viral vector was engineered with a Nano-luciferase reporter gene to quantify effectiveness based on bioluminescence. To silence the target virus, siRNA constructs were designed against the transactivating protein (TrAP) which is responsible for activating the promoter driving the native coat protein transcript. This siRNA was placed in the attenuation vector designed to avoid self-targeting by modifying its own TrAP sequence. Viral infections were initiated in tobacco using Agrobacterium DNA delivery. Measurements at 3, 6, and 9 days post infiltration revealed a significantly reduced luminescence in attenuation treatment demonstrating suppression of the target virus transgene expression. This work sets the stage for a breadth of virus attenuation studies that can be rapidly applied to diverse crop threats.

**Supplementary Information:**

The online version contains supplementary material available at 10.1038/s41598-025-09038-3.

## Introduction

While it is clear that viruses cause billions of dollars in crop damage annually, it is also recognized that this is a predictable consequence of modern agronomic monocultures^1^. The less deleterious, asymptomatic, and even positive role of viruses in the natural environment is increasingly recognized^2,3^. The potential for impact should not be surprising given the episomal, replicative, high copy number characteristics of viruses. For detrimental viruses, avoidance via virus-indexed supply chain, vector control, isolation and plant destruction are major strategies^4^. Genetic engineering of plant immune response such as hypersensitive response can be effective, but are highly specific, require protracted time frames to develop, and may have limited durability as observed for breeding resistance^5^. There is considerable historic research demonstrating that expression of viral genes in transgenic plants can disrupt virus proliferation^6,7^, and defective interfering DNA was developed as an antiviral method long before our understanding of gene silencing^8^. What is clear from these observations is that the expression of viral genes in transgenic plants as well as cross-protection^9^ give insights into the relative ease with which one can disrupt the sophisticated plant-virus relationship.

**Fig. 1:**
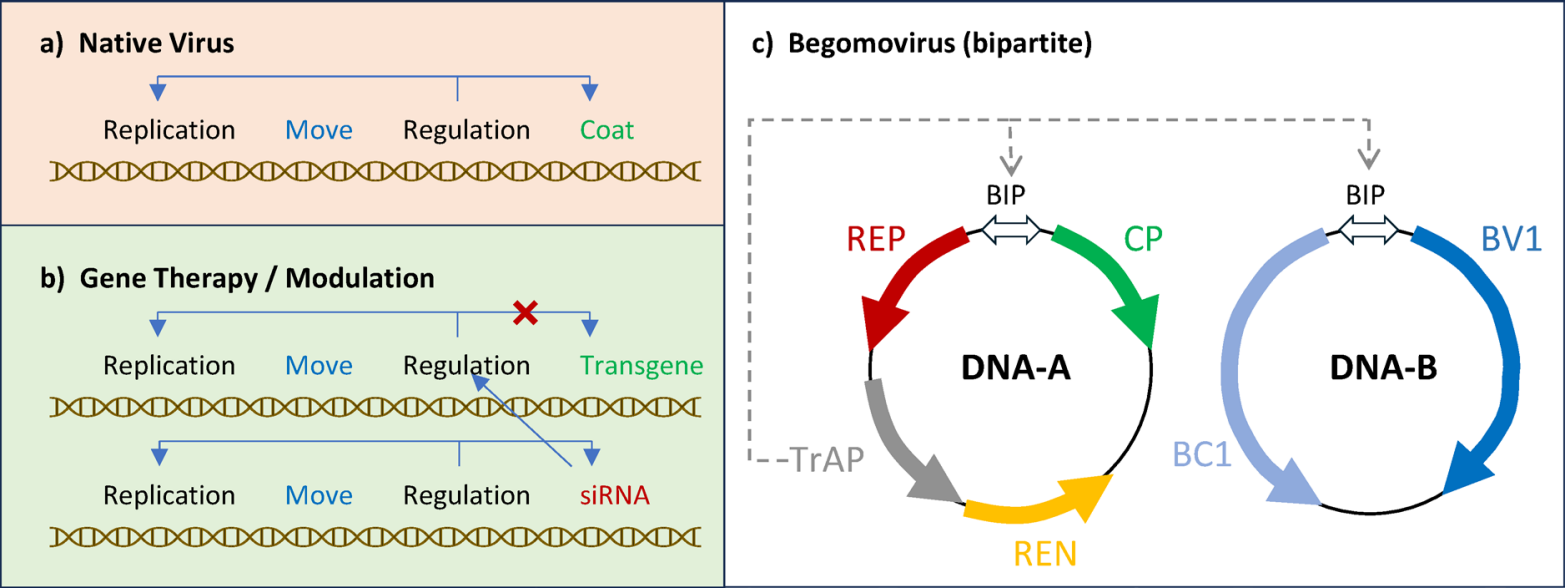
A simplified virus attenuation/gene therapy design based on (**a**) the native viral gene functions which are manipulated to create (**b**) a target protective gene therapy vector by replacing the coat protein, and a corresponding attenuation vector based on gene silencing of the viral regulatory elements. (**c**) Schematic of a bipartite begomovirus (circular ssDNA) where transactivating protein (TrAP) activates the sense strand from the conserved bidirectional intergenic promoter (BIP) for expression of coat protein (CP) and movement (BV1).

The utility of plant viral elements is reflected in the near ubiquitous utilization of viral promoters, UTRs, and subgenomic viral vectors. A major focus of viral vectors has been their use for high-level heterologous expression as a protein production platform^10^, and for virus induced gene silencing^11^. We suggest an alternative application is low-level expression of protective genes that does not require plant transformation. The diversity of plant viruses represents an equally diverse opportunity to introduce episomal DNA for crop protection. There are nine orders of plant viruses including, DNA or RNA, double-stranded or single-stranded, plus- or minus-sense in nature^12^. The basic functions of a native virus are replication, movement, regulation (of life cycle) and encapsidation to facilitate transmission (Fig. 1A). The control of these functions will ultimately determine the success of a protective gene therapy while avoiding the inevitable risks as reflected in human gene therapy^13^. Replication cannot be excessive, or it will undermine plant productivity. Systemic movement needs to be efficient to reduce application load, and biocontainment typically involves manipulation of viral encapsidation by a multifunctional coat protein. Early efforts at attenuating viral symptoms focused on replication^14^ and virus movement^15^. We proposed an attenuation vector design that focuses on regulation (Fig. 1B), where the coat protein is replaced with the transgene, and gene silencing targets the manipulation of the viral regulatory elements.

Geminiviruses encompass the potential of an attenuation vector for reducing detrimental economic impact and/or use for expressing heterologous genes^16,17^. These single-stranded DNA viruses are insect transmitted in nature and are responsible for billions of dollars in damage to crops such as beans, corn and cassava that is disproportionately affecting farmers in developing countries^18,19^. These small DNA viruses (< 10 kb) are well-characterized, and readily manipulated in a plasmid form, as well as launched into replicating single-stranded form using a partial tandem repeat. The begomovirus genus of the family *Geminiviridae* contains bipartite members with increased ease of manipulation and delivery (Fig. 1C). Begomoviruses do not encode their own polymerases and recruit numerous plant proteins to further condense the fundamental virus functions. Another important characteristic of these viruses is a sophisticated persistent insect delivery which provides multiple strategies for biocontainment. An important constraint of these highly condensed viruses is that most of the proteins are multi-functional, where for example the coat protein facilitates nuclear localization as well as participating as a component of the systemic movement complex^20^.

The goal of this research is to test the feasibility of creating a homologous viral vector that is designed to modulate transgene expression from a plant protective viral vector. The mechanisms of plant gene silencing reveal a complexity that matches the diversity of viral strategies to overcome them. Post transcriptional gene silencing (PTGS) responds to aberrantly high levels of a specific transcript – which includes both viral infection as well as transgenes in GMO plants^21^. There are multiple Argonaute / Dicer systems that facilitate the production of small interfering RNAs (siRNA) to accomplish the destruction of target RNA^22^. Some siRNAs can also interact with DNA to interfere with transcription itself through RNA-directed DNA methylation (RdDM)^23^. This provides an alternative strategy of heritable sequence-specific interference that is particularly effective at altering the interactions of transcription factors in epigenetic transcriptional gene silencing^24^.

**Fig. 2:**
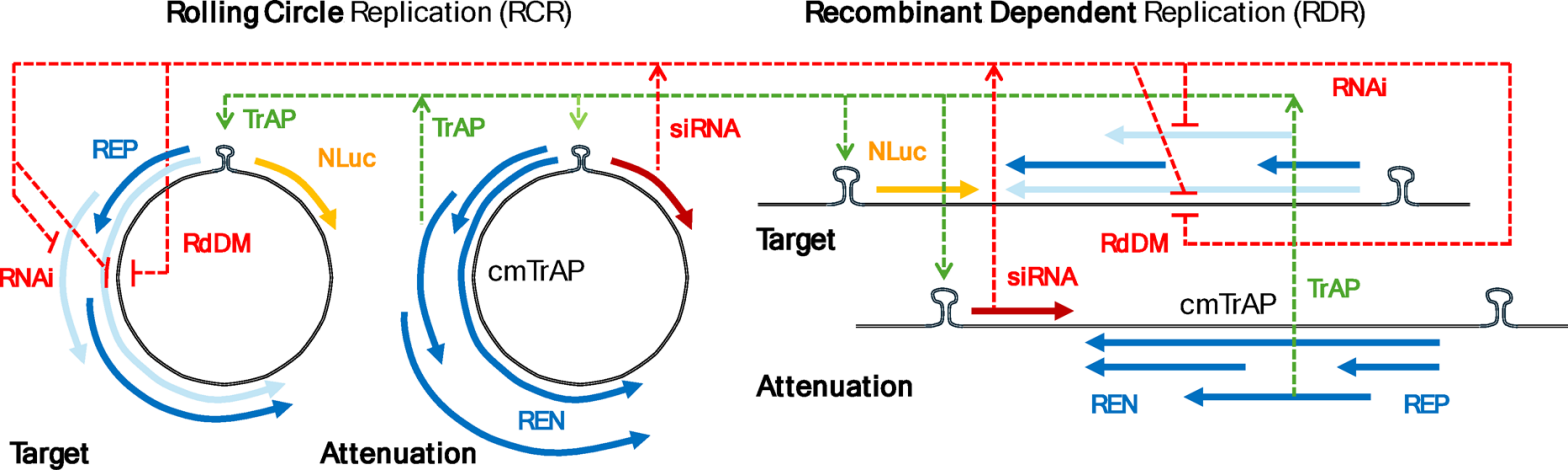
A schematic of the attenuation vector strategy for a bipartite begomovirus. The coat protein is replaced with nanoluciferase (NLuc) for quantitative assessment of attenuation for the Target vector. A silencing small RNA siRNA on the attenuation vector avoids self-attenuation due to a re-coding of the viral sequence for the TrAP gene (cmTrAP). Both the circular and linear replicating forms of the A-component are shown to represent the alternative viral replication modes of rolling circle (RCR), and recombinant-dependent replication (RDR).

The success of the geminivirus class of plant viruses is indicative of their ability to circumvent gene silencing. While there is evidence for anti-gene silencing elements^25^, the efficiency of amplification, and its utilization of critical plant functions limits the targets for silencing. The mechanism of geminivirus amplification (Supplemental section S1) was initially explained via rolling circle replication (RCR)^26,27^. Examination of the amplicons via gel electrophoresis revealed a major component of the replicons as linear heterogeneously primed double-stranded DNA^28^, leading to the confirmation of recombination-dependent replication (RDR). The speed of amplification to thousands of genome copies per cell, in conjunction with efficient systemic movement of these small genomes, can rapidly outpace typical siRNA and RdDM silencing. Taking these observations into account, we have designed a silencing strategy that would ‘piggy-back’ on the replication assets of the virus. Our strategy in developing an attenuation viral vector is to utilize homologous viral functions, to disproportionately affect the target viral vector as shown schematically in Fig. 2.

This figure presents both the circular and linear viral DNA forms that undergo rolling circle replication and recombination-dependent replication. The red dashed lines indicate the regions for sequence specific complementation and associated silencing which are found on the target gene therapy vector, but not the attenuation vector. The green dashed lines represent the interaction of the TrAP protein to activate the sense strand reading frame. Both the target and the silencing vector are based on the A-component of the bipartite begomovirus by replacing the sense strand coat protein reading frame with the NanoLuc luciferase (NLuc) transgene for the target vector, and the silenencing RNA (siRNA) for the attenuation vector. The B-component of the virus was not modified. The TrAP sequence present in the suppression vector was recoded (cmTrAP) to avoid DNA and mRNA silencing while expressing the same TrAP protein. The antisense replication polycistron is known to generate multiple read-through transcripts (AL1-3, AL2-3 and AL3) where only the replication enhancement transcript for REN would not be subject to silencing either through PTGS or RdDM (light and dark blue arrows in Fig. 2). We describe here a proof-of-concept based on the tomato mottle virus (ToMoV) by suppression of NanoLuc luciferase expression in the presence of the attenuation vector. This work sets the stage for the application of this vector system to deliver plant protective genes as well as test the feasibility of attenuation of a native viral infection using this strategy.

## Results

### Target infective clone viral vectors

An agrobacterium binary vector cassette for heterologous transgene expression was created as a 1.5-mer tandem repeat ‘infective clone’ within the T-DNA of the compact pLSU vector: pLSU1//ToMoV{A,1.5-mer}(CP-/transgene payload). This infective clone provided the backbone for the creation of both the attenuation vector by the inclusion of an siRNA targeting TrAP, and the NanoLuc luciferase as the target reporter viral vector. The deconstructed viral reporter contained a synthetic plant codon-optimized version of NanoLuc luciferase^29^ to provide quantitative high-sensitivity assessments of transgene expression. This luciferase reporter gene included an intron from Arabidopsis Argonaute gene (AGO1) to prevent any contribution to bioluminescence from expression in Agrobacterium (pLSU1//ToMoV{A,1.5mer}(CP-/NLuc-I{AGO-405}).

**Fig. 3:**
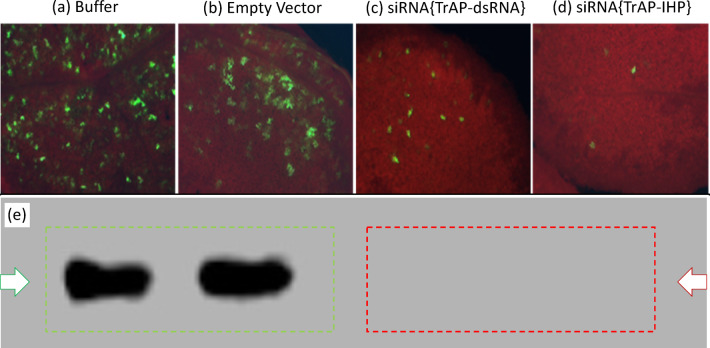
Confirmation of viral silencing of Agrobacterium transient expression of eGFP in the presence of buffer and empty vector (**a** and **b**), and silenced using non-viral transient expression of the dsRNA (**c**) and hairpin (**d**) constructs co-infiltrated with a viral vector, pMon521//PepGMV{A,1.5}(CP-/eGFP +). (**e**) Southern blot probed with GFP chemiluminescence probe; original nitrocellulose membrane from LiCor C-Digit Scanner provided as Supplemental Figure S7.

As a preliminary visual test of the functionality of siRNA for silencing the target viral vector, a target infective clone with eGFP was used as a reporter gene. The infective clone was based on the close ToMoV relative, pepper golden mosaic virus (PepGMV), and using an alternative pMON521 binary vector as these were in hand (pMon521//PepGMV{A,1.5-mer}(CP-/eGFP +), and consistent with the initial priority PepGMV target before constraints with USDA permitting of performance sites resulted in a change to ToMoV. A simple (non-viral) transient expression vector was used to express siRNA based on the PepGMV REP-TrAP-REN antisense strand. As shown in Fig. 3a–b, both the siRNA (dsRNA) and inverted repeat hairpin (IHP) versions were very effective at preventing GFP expression at 3-days post infusion. Large numbers of eGFP fluorescent cells were present in *N. benthamiana* that did not contain siRNA (Fig. 3a–d). Small numbers of eGFP positive cells were present when silenced (Fig. 3c–d). Essentially complete suppression of GFP expression is confirmed in the Southern blot probed with GFP specific chemiluminescence probe, Fig. 3e, where there is no band visible in the presence of either of the siRNA designs.

### Silencing viral vector designs

The attenuation vector included codon modification of its TrAP (AC2) gene (cmTrAP) to avoid self-targeting (pLSU1//ToMoV{A,1.5-mer, cmTrAP}(CP-/siRNA{TrAP}). There are only 47 non-overlapping base pairs of the 390-bp TrAP gene due to the overlap of the AL1-3, REP-TrAP-REN reading frames. With the constraint of only making in-frame codon usage changes to retain the same TrAP protein expression, this resulted in 17 base pair changes as described in the methods and supplemental. The initial focus of non-coding siRNA was based on the intron-splicing hairpin design (IHP) due to its demonstrated superiority for silencing in plants^30^. Although hairpin designs have been reported to be particularly effective based on the production of the dicer-derived targeting components without a need for complementary pairing with the target mRNA sense strand^31^, we found these vectors to be unstable—even in *E. coli*. A rapid mutation of the hairpin vectors was observed in week-long repeated culturing of the inverted hairpin vector (Supplemental section S4).

The TrAP protein is responsible for activation of sense strand promoter activity within the bidirectional stem-loop intergenic promoter region of the native virus; silencing TrAP will therefore attenuate expression of the coat protein on the A-component and nuclear shuttle protein on the B-component of the bipartite begomovirus. More importantly, in addition to silencing the gene therapy coat protein replacement, silencing TrAP will also suppress the expression of the siRNA that is driven by the coat protein promotor – thereby creating a feedback inhibition loop. The TrAP codon modification provides bias against the target viral genome while permitting TrAP expression from the attenuation vector.

### Suppression of the target transgene with homologous viral suppression vector

**Fig. 4:**
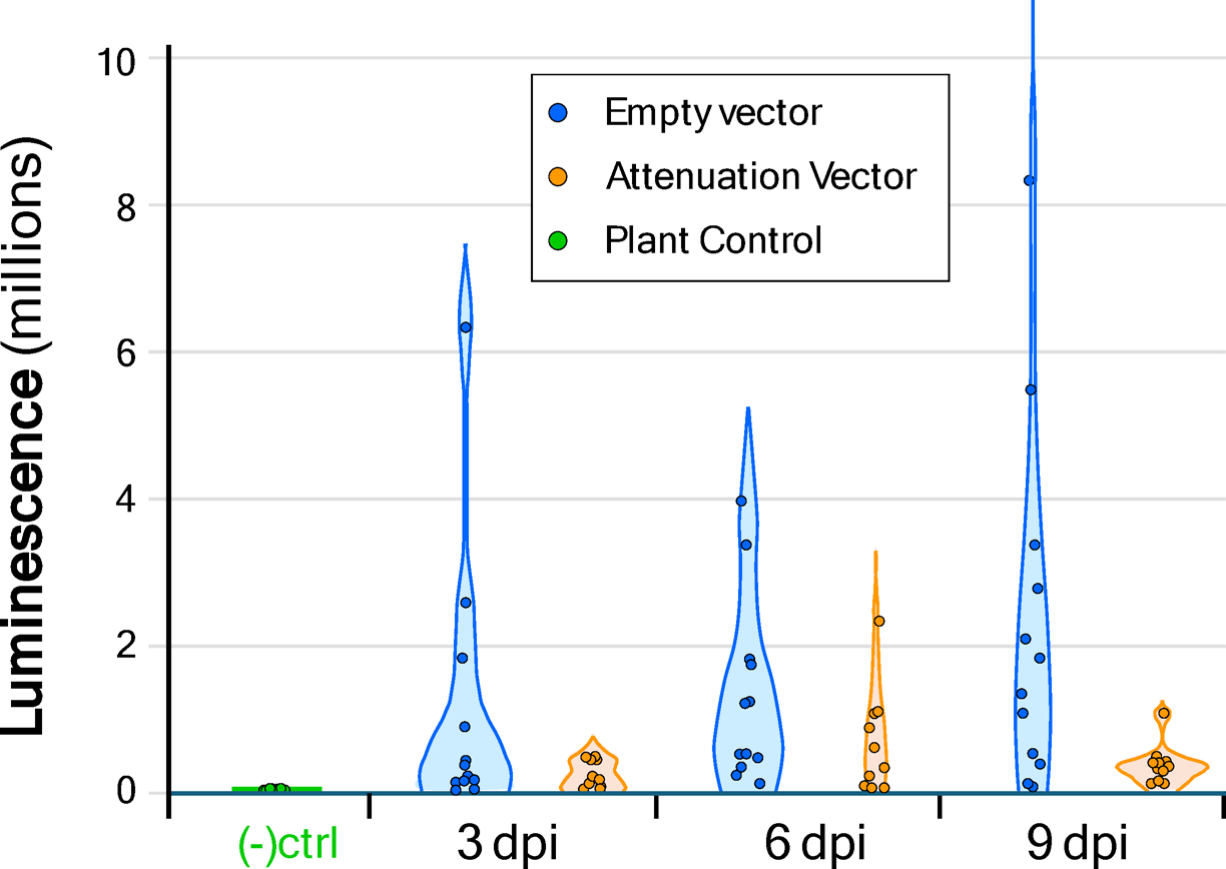
Reduction of luciferase transgene expression with the inclusion of the attenuation vector. Measurements of luciferase activity were measured at 3, 6, and 9 days post agrobacterium co-infiltration (dpi). NanoLuc luciferase activity was normalized to sample total protein content for 3 biological replicate plants, 4 samples taken per plant (total of 12 samples). The results display non-normal distributions which is addressed in the statistical analysis in the Supporting Data file.

The attenuation vector, pLSU1//ToMoV{A,1.5mer, cmTrAP}(CP-/siRNA{TrAP}), was co-infiltrated with the Target vector pLSU1//ToMoV{A,1.5mer}(CP-/NLuc-I) along with a simple coat protein expression vector to provide for nuclear localization and tested for bioluminescence by taking leaf punches and addition of the furimazine luciferin substrate. As observed in Fig. 4, the levels of luciferase for the attenuation vector treatment were much lower than the co-infiltrated empty vector control. The overall average reduction in bioluminescence counts was 94%. The plant control infiltrated only with only empty vector was negligible above background.

These results are consistent with a substantial bias against the target vector, likely representing a displacement by the attenuation vector. A statistical test of the pooled data gives a statistically significant reduction in luminescence (*P* = 0.0015) although the data does not display a normal distribution based on the Kolmogorov–Smirnov Test of normality. Both the Kruskal–Wallis (H = 9.31) and Mann–Whitney (U = 340) non-parametric tests confirm statistically significant reduction in luminescence (*P* = 0.0023). Details of the statistical analysis are provided as a spreadsheet in the Penn State DataCommons archive server and includes an assessment of the kernel density estimation that is the basis of the violin scatter plot.

**Fig. 5:**
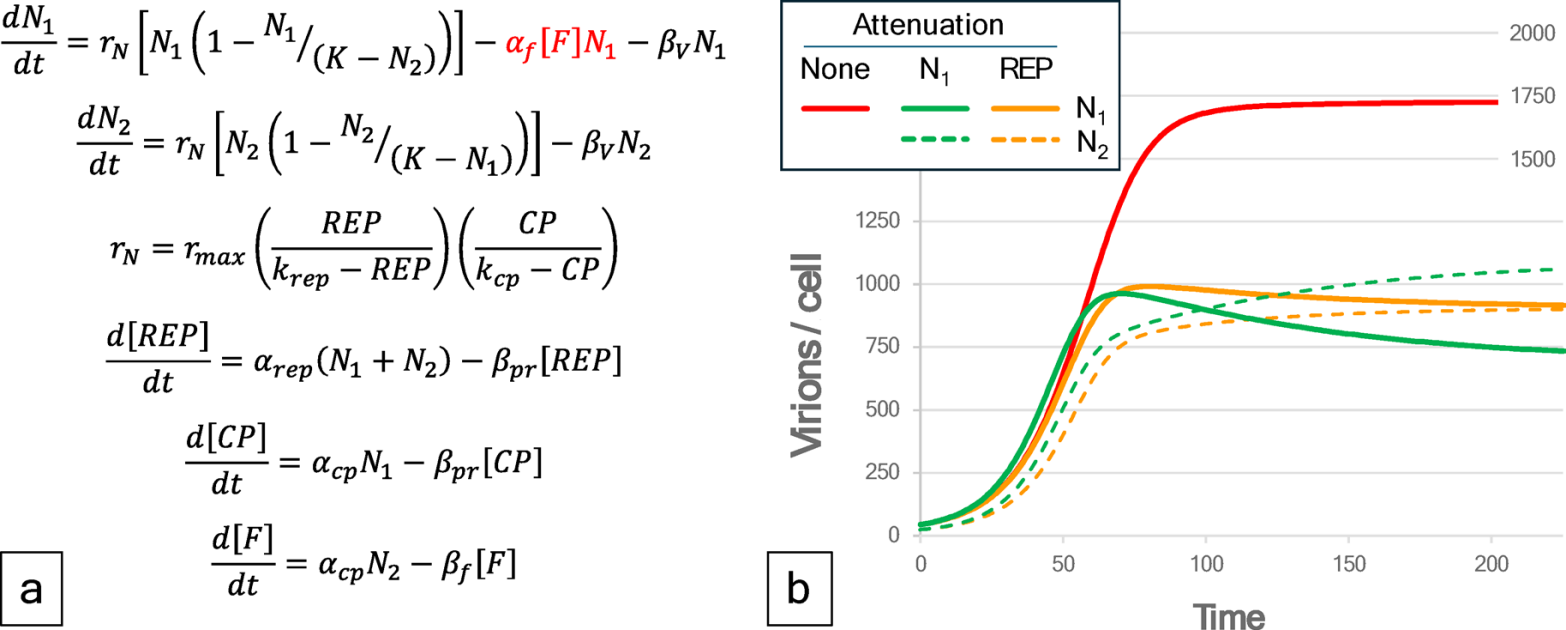
Feedback regulation models provide for quantification and visualization of attenuation vector. (**a**) Simple model of general feedback suppression of target virus (N_1_) based on expression of feedback suppressor [F] encoded on the attenuation viral vector (N_2_). (**b**) Model dynamics for baseline viral proliferation, and two feedback attenuation models that focus on silencing of replication, and general suppression of target virus proliferation. Details of model definition and parameterization are provided in supplementary materials.

Microbial plasmids are the natural analogue of episomal gene therapy, and some organisms such as cyanobacteria are ‘naturally competent’ and readily take up exogenously supplied DNA fragments^32^. Mechanisms to test the utility of exogenous DNA are logical when one considers the unparalleled physiological potential of DNA. An inherent aspect of plasmid gene acquisition is a regulation of plasmid copy number through a negative feedback control loop including antisense inhibition of replication^33^. The research here represents an analogous approach to develop a modulated plant episomal gene acquisition system. Figure 5 presents qualitative modeling of feedback behavior applied to our attenuation virus. See Supplemental section S6 and DataCommons spreadsheet for details.

The feedback functionality can be applied specifically to the target deconstructed therapy virus (N_1_), in which case the attenuation virus (N_2_) can eventually exceed the level of the target. Feedback that more specifically targets the antisense strand REP-TrAP-Ren transcript is predicted to converge to similar virion levels for both target and attenuation virus. Such models provide a basis for hypothesis testing, where the structure of the model (Fig. 5A) is strongly governed by a capacity limit (K) for the viral load being shared between these competing viruses. It can be anticipated that the impact of introducing changes to expression of viral components will be significant and largely detrimental based on observations of expression of viral components in plants. Where early efforts focused primarily on plant protection provided by expressing viral coat protein, those initial observations have been greatly expanded to the expression of other plant viral components^34^. This suggests that the nature of plant viral infections can be extremely sensitive to an imbalance in viral components, and attenuation can be expected to be a relatively common result.

## Discussion

The historical (1968) contribution of plant systems in advancing gene therapy is widely recognized, where the first demonstration of viral gene therapy was the genetic engineering of tobacco mosaic virus (TMV) to introduce a heterologous transgene appended to this RNA virus chromosome^35^. An important distinction in gene therapy approach is whether the gene is episomal or integrated into the chromosome. An extensive focus of initial human gene therapy efforts was based on chromosomal integration such as homologous recombination^36^ or retroviral insertions^37^. The dangers of random insertion are not just the mutation of important genes, but activation of detrimental genes such as those related to cancer. The course of human gene therapy development has subsequently shifted to episomal genetically modified viruses such as lentiviral and adenoviral systems—leading to the first FDA approved human gene therapy being based on these episomal systems^38^.

In contrast, ‘gene therapy’ for crop improvement has evolved entirely to chromosomal integration. Given the ability to make transgenics and counter-select for aberrant phenotypes created during transformation, chromosomal integration has been the focus for crop improvement – particularly with the advent of CRISPR which allows for genetic manipulation based on genomic safe harbor sites^39^. By comparison, human germline gene therapy is not currently an option. The advantage of episomal plant gene therapy with largely asymptomatic viral infection (see Supplemental section S2) is the potential for treatment for biotic and abiotic stresses in the field which is anticipated to become more important due to climate disruption. Combined with precision agriculture enabled by spatial monitoring, this approach can provide for treating crop variability that is inherent in field production. Virus replication and movement become a mechanism for systemic stress treatment, and the methods of transmission dictate considerations for biocontainment.

Biosafety and biocontainment was a major consideration for our selection of begomoviral vectors. Absence of the whitefly vector provides containment by comparison to mechanically transmitted viruses. The specificity of insect-transmitted geminiviruses is illustrative of biocontainment opportunities, where a single amino-acid change in the coat protein can alter gut uptake of the encapsidated virus and prevent persistent transmission^40,41^. Notably, our efforts included trying to design viral vectors that could be insect transmitted based on restrictive size constraints, where the default for genome modifications will prevent encapsidation and transmission. The ability to chromosomally integrate viral functions into the host plant as a basis of complementing the removal of viral genome functionality is also a major future opportunity for biocontainment based on deconstructed viral vectors. Viral genome fluidity is always a concern for the use of viral vectors, where the degrees-of-freedom for geminiviral mutation are severely limited by the multifunctionality of their condensed genomes, and their extensive reliance on plant gene functions – including polymerase activity. Our choice of single stranded DNA viral vector avoids RNA viruses which rely on RNA-dependent RNA polymerases that lack proof-reading and associated propensity for homologous recombination^42^. Interestingly, the new infective clone used in this work has only minor differences (mostly silent mutations) from sequence that that submitted to GenBank 20 years ago despite continuous maintenance on whitefly infected plants. In addition, the coat protein change observed in the new ToMoV clone is for a sequence that has been implicated in improved insect uptake in a related virus^43^.

Herein we have described the logic and preliminary results of creating an attenuation vector, that takes advantage of the co-evolved viral-plant-vector relationship. This provides a means of modulating virus proliferation to minimize the negative impacts on plant health. Interpretation of the mechanism of attenuation awaits more detailed measurements of the molecular entities. A component of the experimentally observed attenuation invariably represents a simple competition for viral replication capacity which can be quantitatively assessed using nonsense coat protein replacements as well as altering the ratio and timing of the launched viral vectors. In addition to the intended application of manipulating expression from a plant-protective viral vector, there is also potential to utilize this approach for anti-viral crop protection by rendering a viral infection asymptomatic. A virus can benefit from plant health; therefore, there is likely a natural evolution towards attenuation that should be kept in mind in developing this approach. In this context, what is needed are studies of symptom attenuation and more importantly the ability of such a technology to improve crop productivity in the presence and absence of biotic or abiotic stress.

## Methods

### Target NanoLuc luciferase reporter vector

To provide a highly sensitive, quantitative target vector, a deconstructed viral launch vector was created by removal of the ToMoV coat protein (CP-) and replacement with NanoLuc luciferase (NLuc +). This was accomplished through the generation of an intermediate payload viral vector based on a partial tandem repeat (1.5mer) which facilitates the initiation of viral genome replication.

*Tomato mottle virus Launch Vector (1.5mer):* Infectious clones of the bipartite tomato mottle begomovirus were generated from updated sequence versions of the monomeric ToMoV DNA {A}-component (FS77) and {B}-component (FS78) cloned into the plasmid pGEMEX-I^44^. For DNA {A-NG}, a 671-bp PCR product was generated using primers {ToMF2} and {ToMR2} using a newly cloned isolate analogous to the FS77 template, restricted with EcoRI + ClaI and cloned into the binary vector pLSU1^45^ restricted with EcoRI + ClaI. The resulting clone (ToMoV-A,0.25) contains a single copy of the intergenic region of ToMoV DNA {A-NG}. PCR amplification using primers {ToMF1} and {ToMR1} with FS77 as template generated a 756-bp product containing the ToMoV coat protein (CP) ORF. The DNA was restricted with ClaI + XhoI and cloned ToMoV-0.25 restricted with ClaI + XhoI to generate the intermediate clone ToMoV-0.55. To generate a full-length genomic fragment FS77 DNA was restricted with ApaI to release a monomeric DNA {A}. After gel isolation the monomer was self-ligated and used as a template for PCR with primers {ToMoVA1.0F} and {ToMoVA1.0R}. The DNA fragment was restricted with XhoI + HindIII and cloned into ToMoV-0.55 restricted with XhoI + HindIII. The resulting construct contains a partial tandem repeat of the ToMoV{A) viral genome (ToMoV{A,1.5mer}) on an *Agrobacterium* binary vector T-DNA creates a replicating vector upon delivery by Agro-infection.

*pLSU1/ToMoV{A,1.5mer}(CP-/{eGFP}* +*)*. eGFP is used to replace the coat protein in the ToMoV-A launch vector. A 720-bp PCR product containing the eGFP ORF was amplified with primers {eGFPF} and {eGFPR} from pGAD-eGFP^46^ as template, restricted with ClaI-XhoI and cloned into the ClaI-XhoI sites of the ToMoV{A,1.5mer} plasmid to generate a transient expression viral vector where eGFP is expressed from the native ToMoV *CP* promoter, ToMoV{A,1.5mer}(CP-/eGFP^+^). This construct was moved into the pLSU-1 binary vector T-DNA by restricting ToMoV{A,1.5mer}(CP^-^/eGFP^+^} with HindIII-EcoRI to release the corresponding respective 3300-bp fragment containing the corresponding 1.5mer elements. The DNA fragments were then cloned into the binary vectors pLSU1^46^ at the HindIII-EcoRI sites. The compact, high-copy number pLSU Agrobacterium binary vector backbone was kindly provided by Martin Schattat at Martin Luther University Halle-Wittenberg. pLSU1 is designed for transient expression and lacks a plant selectable marker.

*pLSU1/ToMoV{A,1.5mer}[CP-/(NLuc)* +*].* The tomato codon-optimized NanoLuc Luciferase was chemically synthesized based IDT tool, https://sg.idtdna.com/pages/tools/codon-optimization-tool. For construction of a ToMoV{A,1.5mer}-component viral vector with the 516-bp NLuc, the payload NLuc gene sequence was amplified by PCR from pLSU1/t35s:NLuc:NosT with 5’ ClaI and 3’ XhoI restriction site extensions using primers {ToNLucF} and {ToNLucR} and digested using ClaI and XhoI restriction enzymes. The gel extracted NLuc gene was then ligated with similarly digested pLSU1/ToMoV{A,1.5mer}[CP-/{mNG-I} +]. The ligation was transformed into competent *E. coli* Top-10, creating the pLSU1/ToMoV {A,1.5mer}[CP-/NLuc +]. Addgene = 224948.

*pLSU1/ToMoV{A,1.5mer}[CP-/(NLuc-I{AGO-405}* +*)].* The intron sequence chosen from the Arabidopsis Argonaute (AGO) gene was chemically synthesized from TWIST BioScience. NLuc gene and AGO intron gene was then PCR amplified with overlap primers {(Z1nLuc)/(Z2nLuc )} & {(Z5nLuc)/(Z6nLuc)} and {(Z3nLuc)/(Z4nLuc)} respectively. The obtained fragments were then assembled by Fusion PCR and subsequently cloned into ToMoV vector to create pLSU1/ToMoV{A,1.5mer}[CP-/(NLuc-I{AGO-405}) +]. Addgene = 224951.

### Viral vector silencing with non-viral siRNA expression vectors

Preliminary testing of the effectiveness of deconstructed viral vector silencing was undertaken with an analogously generated deconstructed pepper golden mosaic virus (PepGMV) infective clone expressing enhanced GFP (eGFP) as a replacement for the coat protein (pMon521//PepGMV{A,1.5}(CP-/eGFP +). Both simple siRNA and hairpin RNA were created to test general silencing using the agrobacterium binary vector cloning vector pFGC1008 (GenBank = 32351265, available through the Arabidopsis Biological Resource Center, stock number CD3-446). Silencing was implemented using a fragment of AL1-AL3 expressed under the 35S promoter in the agrobacterium binary vector, pFGC1008. This preliminary work was conducted with the pepper golden mosaic virus (PepGMV) at the early stages of the DARPA project when this was the viral vector focus of the contract. This PepGMV infectious clone vector was created analogous to the ToMoV deconstructed viral vectors described above, only starting with a pepper golden mosaic virus 1.5-mer infective clone. The PepGMV genome fragment used to target the infectious virus consists of nt1696 to nt1202 containing sequences homologous to the AL1, AL2 and AL3 genes. The single copy (pFGC1008-PepALforward) will produce a virion sense small RNA. The inverted repeat (pFGC1008-dsPepAL) will produce an RNA capable of forming a stem-hairpin with a PepAL stem and a GUS loop dsRNA and is expected to be a substrate for DICER and RISC to generate siRNA targeting AL1, AL2 and AL3. Total DNA isolated 3-days post-infiltration from Agro-infused *N. benthamiana* leaves was run on an agarose gel and probed with GFP chemiluminescence probe (Southern blot) on a nitrocellulose membrane using a LiCor C-Digit scanner.

### Non-self targeting attenuation vector

This attenuation vector is based on a deconstructed tomato mottle virus (ToMoV) payload pLSU1/ToMoV{A,1.5mer}(CP-/payload). In this deconstructed virus, endogenous CP is replaced with a 390-bp antisense silencing construct (siRNA native TrAP gene in antisense orientation). The siRNA was amplified with primers {siTrAPF} and {siTrAPR} to generate ClaI and XhoI overhangs, restricted with ClaI-XhoI and cloned into the ClaI-XhoI sites of the TrAP codon-modified ToMoV infective clone to generate binary vector. Note that REP-TrAP-REN are overlapping cistrons that are transcribed as both tandem and individual mRNA. The codon modification (IDT.com) focused on the 47-bp non-overlap TrAP sequence while maintaining expression of the same TrAP protein. The codon modified TrAP gene (cmTrAP) sequence in the 1.5mer was synthesized from TWIST Bioscience. The obtained sequence was PCR amplified using PCR primers {Co-1.5mer-F} and {Co-1.5mer-R} to generate XhoI and HindIII overhangs, followed by restriction digestion and ligation into the XhoI—HindIII digested pLSU1/ToMoV{A,1.5mer}(CP-/ siRNA{TrAP}) and further transformations in competent *E. coli* Top-10 generating pLSU1/ToMoV{A,1.5mer,cmTrAP}(CP-/ siRNA{TrAP}) clones. The sequence was confirmed by Sanger sequencing based on the forward primer {Co-1.5mer-F}. Additional cloning details can be found in (Supplemental sections S3 and S5).

### Coat protein (CP) transient expression vector

Since the coat protein has important functions of nuclear localization and the viral movement complex in addition to the implied role of virion encapsidation, this must be supplied *in trans* for deconstructed CP- viral vectors. Although we have generated CP + transgenic plants as a means of complementation to achieve viral containment (unpublished), this initial demonstration utilized the inclusion of a simple coat protein transient expression vector created by insertion into the multiple cloning site (MCS) of our pLSU1 transient expression ‘drop in’ vector (pLSU1/t35s[TMV]:MCS + :NosT (Addgene #212161) to create pLSU1/35s:CP{ToMoV}.

### Transient expression/agrobacterium infiltration

Agrobacterium transient expression was based on syringe infiltration procedure^47^. Binary vectors were introduced into our Cys32 cysteine auxotroph strain of Agrobacterium (C58 chromosomal background, EHA105 helper plasmid)^48^. Agrobacterium containing the binary vector was grown from cryostock on selective LB solid media, and then grown overnight to an OD_600_ < 1.0 on liquid media. The liquid culture was pelleted and resuspended to an OD_600_ = 1 in an infiltration buffer containing 10 mM MES and 10 mM MgCl_2_. Incubation for 1-h at 28 °C after inclusion of 0.1 mM acetosyringone to activate T-DNA transfer. Surfactant 0.02% Silwet L-77 was added to facilitate gentle syringe infiltration through the stomata on the underside of 4-week old *N. benthamiana* plants that were grown with 12-h photoperiod. Plants are hardened at room humidity for at least 24 h to reduce necrosis.

### Assay for NanoLuc luciferase (NLuc)

Leaf tissue was taken with a #8 (11 mm) punch from infiltrated plants. Tissue samples were placed in clean sterile bead beater tubes and frozen in liquid nitrogen before immediately grinding in a Minibeadbeater-24 (BioSpec) at 2400 rpm for 8 s. Freezing/beating process was repeated until the sample was visually uniformly finely ground followed by an immediate addition of CCLR extraction buffer (Promega, Luciferase Cell Culture Lysis Reagent, cat. #E1531) at 2 mL per g fresh weight. The plant sample was then centrifuged at 3000 g for 30 s in a table-top centrifuge. Dilutions of plant samples from 40- to 1000-fold were made and run through a Bradford assay with BSA concentrations of 0.001–0.025 µg/µL used as a reference. The 400-fold dilution of each sample was within the BSA standard curve, and this was used as the relative total soluble protein concentration when normalizing the luminescence data. Luminescence was measured by taking 5 µL of leaf extract supernatant with 5 µL of NLuc luciferin reagent (furimazine + buffer, Promega, cat. #N1110) for measurement of bioluminescence in a TECAN Infinite 200 PRO. Integration time on the TECAN was set to 10 s.

### Instability of hairpin in *E. coli*

Despite initial sequence confirmations and utilizing RecA- cloning strains (NEB Stable, cat. #C3040I) subsequent resequencing of the plasmids after 5 serial passages revealed that these hairpin siRNA vectors were prone to recombination. In addition, it was found that plasmid sequencing would consistently fail which required sequence confirmations based on PCR amplifications. PCRs were performed for the hairpin (sense:intron:antisense) as fragments spanning the intron region and the hairpin extremes at 5′ sense and 3′ antisense regions {(TN1F / TN2R) and (TN3F / TN4R) with amplicon length of 304 and 378 bp respectively}. Repeated testing confirmed the presence of sense strand alone. Antisense strand was speculated to have undergone recombination (see Supplemental section S4). Due to this instability, the hairpin design was not carried out through further testing.

### Attenuation of viral vector expressing nano-luciferase

To demonstrate the effectiveness of the ssRNA attenuation viral vector, an experiment was performed to reduce NanoLuc luciferase expressed from the viral target pLSU1/ToMoV{A,1.5mer}[CP-/(NLuc-I{AGO-405}+)] viral vector. The attenuation treatment included an equal volume of pLSU1/ToMoV{A, cmTrAP,1.5mer}(CP-/siRNA{TrAP}). A control treatment included an empty binary vector (pLSU1/t35s:[TMV]:MCS:nosT ; Addgene #212160) instead of the attenuation vector. To facilitate nuclear localization and systemic movement, both treatments included co-infiltration with ToMoV coat protein (pLSU1//35s:CP{ToMoV}:NosT). All constructs were added in a (1:1:1) ratio based on an equal OD and then equal volume amounts. Additionally, a plant control with no infiltration was included as well as an infiltration buffer control which contained no agrobacterium constructs. These treatments were vacuum infiltrated at 25 mm-Hg for 5 min. Three plants were infiltrated per treatment, and four samples per plant (two each from the two best infiltrated leaves) were taken for a total of 48 samples per time point assayed at 3, 6, and 9 days post inoculation (dpi); three samples were lost during cryovial failure (see DataCommons). Protein levels were measured from liquid nitrogen flash frozen (#4 punch) leaf samples extracted using Cell Culture Lysis Reagent (CCLR, Promega) based dilution curves of the Bradford assay. Luminescence was normalized by protein concentration in extract.

### Approvals

ToMoV virus activation via *Agrobacterium* infective T-DNA clones was executed under USDA-APHIS-BRS permit # 19–235-102m. Virus protocols were approved by The Pennsylvania State University Institutional Biosafety Committee.

## Electronic supplementary material

Below is the link to the electronic supplementary material.

Supplementary Material 1.

## Data Availability

The datasets generated and analyzed during the current study are available in the datacommons@psu repository, 10.26208/RM1W-V277. This dataset contains a supplemental spreadsheet file for figure data, statistical analysis and modeling. Viral plant pathogens can be provided under a USDA-APHIS permit.
