# Supplementary material for: A geminivirus attenuation vector for crop protection using episomal plant gene therapy
Source: Sci Rep. 2025 Jul 11;15:25085. doi: 10.1038/s41598-025-09038-3 (PMC12254371; doi:10.1038/s41598-025-09038-3)
Supplement: Supplementary file 1 — Supplementary Material 1. [file 41598_2025_9038_MOESM1_ESM.pdf]

A Geminivirus Attenuation Vector for Crop Protection using Episomal Plant Gene Therapy

Natalie Thompson, Rekha Kandaswamy, Aliya Fathima Anwar, Jane Polston, Garry Sunter, Wayne R. Curtis

SUPPLEMENTARY MATERIAL Index:

S1: Begomovirus Alternative Replication Mechanisms
S2: Comparison of the Whitefly / ToMoV Symptoms in Tomato
S3: Important sequences / Alignments for the Construction of the Attenuation vector:
S4: Generation and Instability of the Viral Attenuation Vector hairpin siRNA
S5: Sequences and Additional Cloning Details:
S6: Modeling of Attenuation Vector Feedback Modulation
S7: Silencing of eGFP Viral vector – Original Southern Blot for Figure 3e

<u>S1: Begomovirus Alternative Replication Mechanisms</u>: Begomovirus replication is illustrated in **Figure S1**, where two mechanisms contribute to generating additional single stranded DNA (ssDNA) as circular viral genomes that are encapsidated for insect transmission. After the replication initiator protein (REP) recruits the plant DNA polymerase-δ and associated viral replisome, this replication complex binds to the conserved intergenic bidirectional intergenic stem-loop promoter region (BIP), depicted in figure as a small stem loop) where it nicks the double-stranded DNA to initiate replication.

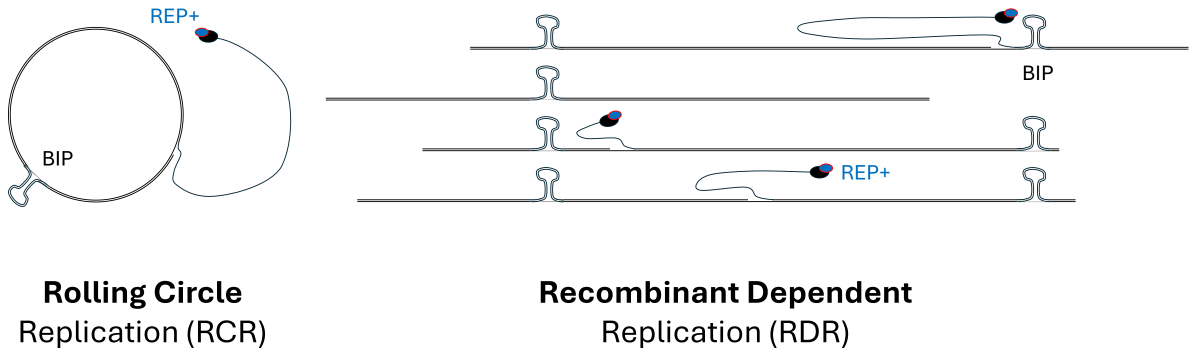

Figure S1: A schematic illustration of the begomovirus replication strategy that is based both on rolling circle replication (RCR) in which the double-stranded DNA is primed at the bidirectional stem-loop intergenic promoter (BIP) by the REP protein to initiate a nicking and sense-strand amplification. Recombinant dependent replication (RDR) results from a more random initiation of double stranded DNA amplification which then serves as a linear template for ssDNA initiation using the same REP-induced nicking and amplification; notably it is this circular single stranded DNA form that is encapsidated for subsequent insect transmission.

<u>S2: Comparison of the Whitefly / ToMoV Symptoms in Tomato</u>:

The tomato mottle virus (ToMoV) was chosen for this study based on observations of minimal detrimental symptoms. A tomato colony with and without the ToMoV (A+B) was established in whitefly insect cates from the wild-type viral components launched using agro-infection of 1.5-mer 'infective clones' in *N. benthamiana* plants, and then transitioned to tomato over a 3-month period with monthly addition of 1-month old tomato seedlings. The presence and absence of virus was confirmed by PCR. The colonies were washed down after 10-min exposure in a cold-room on a roughly 2-week interval to control the whitefly population. Little to no symptoms were observed do to viral infection in the initial month of viral infection relative to virus-free whitefly control.

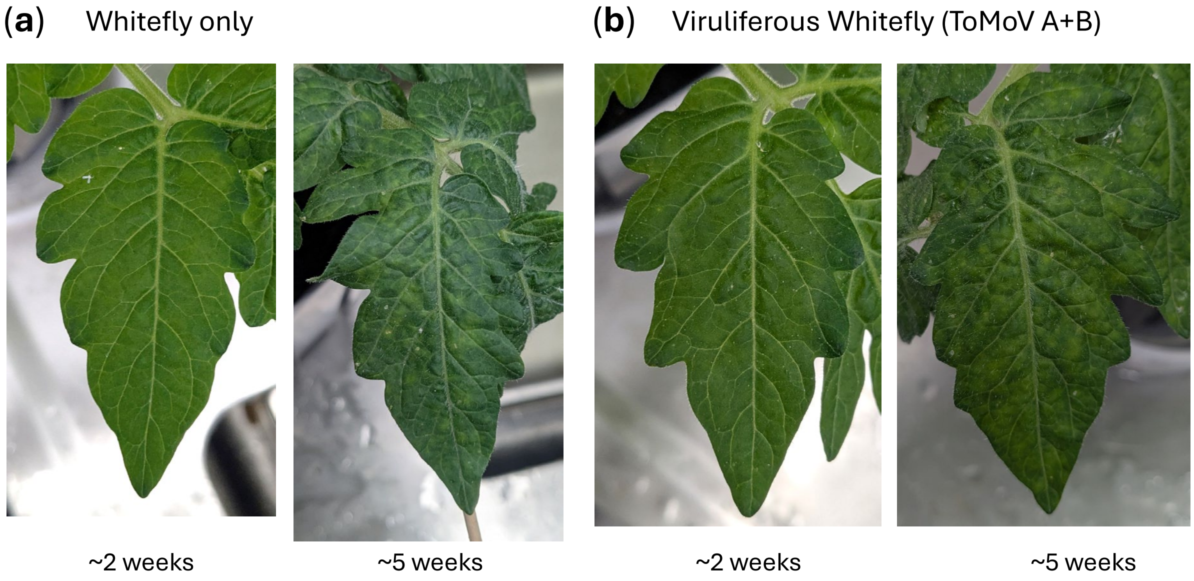

Figure S2: Comparison of symptoms of whitefly infected and viruliferous ToMoV (A+B) whitefly infected tomato (Florida Lanai) plants. (**a**) terminal primary leaflet on 4-5 fully expanded leaf from shoot tip at 2 and 4 weeks after introducing 1-monty old seedlings into the insect cage. (**b**) comparable leaf pictures for viruliferous whitefly colony that is PCR positive for ToMoV A- and B-components.

S3: Important sequences / Alignments for the Construction of the Attenuation vector:

Since tomato mottle virus (ToMoV) is a single stranded DNA, care must be taken to correctly identify the siRNA sequence. The transactivating protein, TrAP is on the complementary virion strand and has significant overlap with both the REP (AL1) and REN (AL3) coding sequences as shown in **Figure S2**. The targeted siRNA therefore targets the non-overlapping region of the TrAP mRNA as given below.

(**a**) ToMoV TrAP Coding Sequence on Complementary Virion Strand with overlapping (REP - REN)

```
ATGCGATCTTCATCACCCTCACAGCCCCCCTCTATCAAGAGAGCACACAGGCAAGCCAAGAAACGGGC
AATCAGAAGGCGCAGGGTTGATCTACAGTGCGGGTGCTCCATCTACTTCCACTTAGACTGTGCGGGAC
ATGGATTCACGCACAGGGGAACTCATCACTGCACATCAGGCGGAGAATGGCGTGTATATCTGGGAGCT
AGAAAATCCCCTTTATTTCAAGATACACAGAGTAGAGGACCCACTGTATACCAGAACGAGGGTATACC
ACGTACAAATACGGTTCAACCACAACCTGAGGAAAGCGTTGCATCTCCACAAAGCCTACCTGAACTTC
CAAGTTTGGACGACGTCGATGACAGCTTCTGGATCAATTTATTTAGCTAG
```

(**b**) Alignment of the Non-Overlapping TrAP with Related Sequences

```
Codon-modified TrAP:          TTTGCAATGTGGTTGTAGTATTTATTTTCATCTTGATTGCGCCGGCC
                              * * ** ** ** **    ** ** ** **  * ** ** ** ** *
Native TrAP (Non-overlap) :   TCTACAGTGCGGGTGCTCCATCTACTTCCACTTAGACTGTGCGGGAC
                              |||||||||||||||||||||||||||||||||||||||||||||||
mRNA TrAP:                    UCUACAGUGCGGGUGCUCCAUCUACUUCCACUUAGACUGUGCGGGAC
                              :::::::::::::::::::::::::::::::::::::::::::::::
siRNA TrAP:                   AGAUGUCACGCCCACGAGGUAGAUGAAGGUGAAUCUGACACGCCCUG
                              :::::::::::::::::::::::::::::::::::::::::::::::
Virion ssDNA TrAP:            TCTACAGTGCGGGTGCTCCATCTACTTCCACTTAGACTGTGCGGGAC
```

Figure S3: Important sequences / alignments for the construction of the attenuation vector: (**a**) Identifying the sequences within the ToMoV TrAP gene that do not overlap with the adjacent 5'-REP and 3'-REN genes. (**b**) Sequences that define the silencing RNA (siRNA) as well as the virion ssDNA form, noting that the REP-TrAP-REN operon is encoded on the complementary virion sense strand.

S4: Generation and Instability of the Viral Attenuation Vector hairpin siRNA:

Considerable effort initially focused on the intron-spliced hairpin siRNA design based on its reported superior performance for silencing in plants and plant viruses. The general notation for the hairpin infective clone design is pLSU//ToMoV{A-NG,cmYYY,1.5mer}(CP-/IHP-xxx). The basic designs targeted both N- and C-terminal regions of the REP and TrAP transcripts where the target sequences were determined bioinformatically while obtaining the desired payload length to maintain a viral genome size close to the ToMoV A-component of 2601-bp (https://doi.org/10.1099/0022-1317-73-12-3225). Maintaining the deconstructed attenuation vector design was predicated on the plan for insect transmission which requires genome size-dependent encapsidation. While creating the attenuation vector constructs, control payload vectors with and without the codon modified TrAP versions of the target genes as payload were also generated – with a plan for more sophisticated assessment of kinetics that would include attenuation vector self-targeting. The rationale for the generation of these (unfortunately) unstable designs is included below for reference.

The intron-spliced hairpin (IHP) siRNA centered around the non-overlapping TrAP gene was found to be unstable. An experiment was undertaken to repeatedly passage *E. coli* harboring this plasmid to assess instability by sequencing. Primers used for sequencing include {TN1F}, {TN2R}, {TN3F}, and {TN4R} as given in **Supplemental Table S5**.

(a)

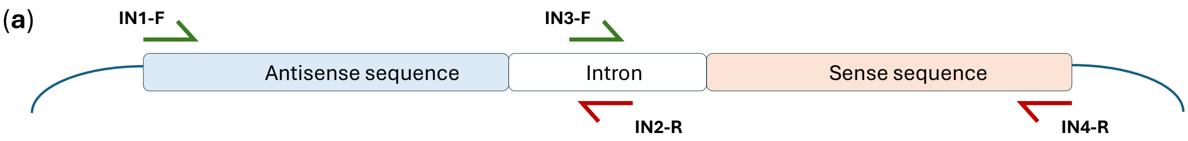

(b)

```
B6400B_PIF     44 ---AT--TTCGTGTGTCTATGATGATGATGATGTTACAG---------------------
B6400B_PAL3R   33 ---AT--TTCGTGTGTCTANGANGATGATGATGTTACAG---------------------
TNB6F2_IF      42 ---AT--TTCGTGTGTCTATGATGATGATGATGTTACAG---------------------
TNB6F2_PAL3R  120 TTGATCAGTCGCTCGTC------GATGATTTTGTTACCGTTCCACAAAGTGCCGGTAACT

B6400B_PIF     78 ------------CCATGGATATAATAGATCTATGCTCAAT---------TCGTCA-----
B6400B_PAL3R   67 ------------CCATGGATATAATAGATCTATGCTCAAT---------TCGTCA-----
TNB6F2_IF      76 ------------CCATGGATATAATAGATCTATTATATT---------GTCGACA-----
TNB6F2_PAL3R  174 GTTATCTTCTTNCCATCG----AAGACAGCAATGNCCTNGTAGGGACGGCCGACGTAGTC
```

Figure S4: Mutations in the intron-spliced hairpin siRNA construct in serial passage. (**a**) Schematic depicting the primers used for sequencing the intron-spliced hairpin. (**b**) The cloned sequence (bottom row) compared to three independent sequencing results after five culture passages (one week) of passaging the binary vector, pLSU1//ToMoV{A,1.5mer}(CP-/siRNA{TrAP-IHP}. The dsRNA design did not display any mutations (not shown).

Rationale for IHP siRNA Designs: The following are IHP constructs targeting both TrAP (AL2) and REP (AL1) as well as several non-silencing controls, designed as the intron-spliced inverted hairpin (IHP) attenuation infective clones. This effort was based on the superiority of the IHP design (Smith et al., 2000, https://doi.org/10.1038/35030305; Wesley et al., 2001, https://doi.org/10.1046/j.1365-313X.2001.01105.x) observed for silencing the AC4 gene of begomoviruses (Praveen et al., 2010; https://doi.org/10.1007/s11248-009-9291-y) These are included since their design might be implemented utilizing RecA- cloning strains.

1. pLSU1 ToMoV{A-NG,1.5mer}(Cp-/IHP-TrAP-C), 7689 bp
In this deconstructed virus, endogenous CP is replaced with a siRNA construct (Intron-hairpin TrAP gene, IHP-TrAP-C) targeting the TrAP gene of payload carrying deconstructed virus. This construct comprises of 210 bp of the C-terminal region of the TrAP gene with an aim to silence the functioning of the TrAP gene. Since the 1.5mer contains the native TrAP sequence, it will also self-silence.

2. pLSU1 ToMoV{A-NG, **cmAL2**-C,1.5mer}(Cp-/IHP-TrAP-C), 7689 bp
In this deconstructed virus, endogenous CP is replaced with a siRNA construct (Intron-hairpin TrAP gene, IHP-TrAP-C) targeting the TrAP gene of payload carrying deconstructed virus. This construct comprises of 210 bp of the C-terminal region of the TrAP gene with an aim to silence the functioning of the TrAP gene. To overcome the limitation of self-silencing, the 210 bp C-terminal target of native TrAP gene is replaced with a codon modified TrAP gene (cmAL2-C, chemically synthesized).

3. pLSU1 ToMoV{A-NG, **cmAL2**-C,1.5mer}(Cp-/miR3A), 8077 bp
In this deconstructed virus, C-terminal region of the TrAP (AL2) gene in the 1.5mer infective clone is modified to serve as vector control for the above construct (#4) and to check the effectiveness of the modified codons in performing the function of the native viral genes. This construct comprises of 210 bp codon modification of C-terminal region of the TrAP gene (cmAL2-C, chemically synthesized) which is a target for silencing TrAP expression. In principle this will generate the same TrAP protein function and not alter the function of the amiRNA payload.

4. pLSU1 ToMoV{A-NG,1.5mer}(Cp-/IHP-TrAP-N), 7622 bp
In this deconstructed virus, endogenous CP is replaced with a siRNA construct (Intron-hairpin TrAP gene, IHP-TrAP-N) targeting the TrAP gene of payload carrying deconstructed virus. This construct comprises of 180 bp of the N-terminal region of the TrAP gene with an aim to silence the functioning of the TrAP gene. Since the 1.5mer contains the native TrAP sequence, it will also self-silence.

5. pLSU1 ToMoV{A-NG,**cmAL2**-N,1.5mer}(Cp-/IHP-TrAP-N), 7622 bp
In this deconstructed virus, endogenous CP is replaced with a siRNA construct (Intron-hairpin TrAP gene, IHP-TrAP-C) targeting the TrAP gene of payload carrying deconstructed virus. This construct comprises of 180 bp of the C-terminal region of the TrAP gene with an aim to silence the functioning of the TrAP gene. To overcome the limitation of self-silencing, the 180 bp N-terminal target of native TrAP gene is replaced with a codon modified TrAP gene (cmAL2-N, chemically synthesized).

6. pLSU1 ToMoV{A-NG, **cmAL2**-N,1.5mer}(Cp-/miR3A), 8077 bp
In this deconstructed virus, N-terminal region of the TrAP (AL2) gene in the 1.5mer infective clone is modified to serve as vector control for the above construct (#5) and to check the effectiveness of the modified codons in performing the function of the native viral genes. This construct comprises of 180 bp codon modification of the N-terminal region of the TrAP gene (cmAL2-N, chemically synthesized) which is the target for silencing TrAP expression. In principle this will generate the same TrAP protein function and not alter the function of the amiRNA payload.

7. pLSU1 ToMoV{A-NG,1.5mer}(Cp-/IHP-REP-C), 8467 bp

In this deconstructed virus, endogenous CP is replaced with a siRNA construct (Intron-hairpin Rep gene, IHP-Rep-N) targeting the Rep gene of payload carrying deconstructed virus. This construct comprises of 330 bp of the C-terminal region of the Rep gene with an aim to silence the functioning of the Rep gene.

8. pLSU1 ToMoV{A-NG, **cmAL1**,1.5mer}(Cp-/IHP-REP-C), 8467 bp
In this deconstructed virus, endogenous CP is replaced with a siRNA construct (Intron-hairpin REP gene, IHP-REP-C) targeting the REP gene of payload carrying deconstructed virus. This construct comprises of 330 bp of the C-terminal region of the REP gene with an aim to silence the functioning of the REP gene.

9. pLSU1 ToMoV{A-NG, **cmAL1**,1.5mer, }(Cp-/miR3A), 8077 bp
In this deconstructed virus, C-terminal region of the REP (AL1) gene in the 1.5mer infective clone is modified to serve as vector control for the above construct (#6) and to check the effectiveness of the modified codons in performing the function of the native viral genes. This construct comprises of 330 bp codon modification of the C-terminal region of the REP gene (cmAL1, chemically synthesized) which is a target for silencing REP expression. In principle this will generate the same REP protein function and not alter the function of the amiRNA payload.

To overcome the limitation of self-silencing, the sequence of native REP/TrAP gene were replaced with a codon modified sequence (chemically synthesized) in the constructs 2, 4, and 6. Additionally three constructs (3, 6, and 9) was created to test the effects of modified viral backbone on the generality of the functional role of the payload such as the effect from viral load capacity in the virus proliferation modeling (see **Supplemental Section S6**).

S5: Sequences and Additional Cloning Details:

Table S5: Primers referenced in the paper.

ToMF2	GCGGAATTCGCCGTCGATTTGGAAATC
ToMR2	GCGATCGATTTAAAGACATTGGGCCAAG
ToMF1	GCGATCGATATGCCTAAGCGTGATTTG
ToMR1	GCGCTCGAGTTAATTCGTGATCGAATC
ToMoVA1.0F	GCGCTCGAGTAAAATTTGAATTTTATTGAATG
ToMoVA1.0R	GCGAAGCTTTTTGAGTTAAAGACATTGGGCCAAG
eGFPF	GCGATCGATATGGTGAGCAAGGGCGAGGAGCTG
eGFPR	GCGCTCGAGTTACTTGTACAGCTCGTCCATGCCGAG
ToNLucF	ACTGCCATGGTGTTTACTCTTGAAG
ToNLucR	ATCGCTCGAGTTAAGCTAATATACGCTCGCA
nLx1/Z1nLuc	TAATATCGATATGGTGTTTACTC
Z2nLuc	ATGAAACTTACCGTTCCACAAAGT
Z3nLuc	ACTTTGTGGAACGGTAAGTTTCAT
Z4nLuc	GATTTTGTTACCTAATTCAGA
Z5nLuc	TCTGAATTAGGTAACAAAATC
nLx2/Z6nLuc	AGATCTCGAGTTAAGCTAATATA
siTrAPF	NNNNNATCGATCTAGCTAAATAAATTGA
siTrAPR	NNNNNCTCGAGATGCGATCTTCATCAC
Co-1.5mer-F	CTCGAGCTCGAGTAAAATTTG
Co-1.5mer-R	CTGGAGTACCCCTTCTTATTA
TN1F	ACTAATCGATTGATCATCCGCC
TN2R	CCAATCAATTAAAAAAATAGATCAG
TN3F	CTGATCTATTTTTTAATTGATTGG (same as TN2F reverse orientation)
TN4R	AATCATGTATTGGAGAATCATTCA

Antisense -TrAP sequence (ToMoV)

PCR primer is underlined which includes the restriction enzyme overhang for cloning into the Agrobacterium infective clone.

ATCGATCTAGCTAAATAAATTGATCCAGAAGCTGTCATCGACGTCGTCCAAACTTGGAAGTT
CAGGTAGGCTTTGTGGAGATGCAACGCTTTCCTCAGGTTGTGGTTGAACCGTATTTGTACGT
GGTATACCCTCGTTCTGGTATACAGTGGGTCCTCTACTCTGTGTATCTTGAAATAAAGGGGAT
TTTCTAGCTCCCAGATATACACGCCATTCTCCGCCTGATGTGCAGTGATGAGTTCCCCTGTGC
GTGAATCCATGTCCCGCACAGTCTAAGTGGAAGTAGATGGAGCACCCGCACTGTAGATCAA
CCCTGCGCCTTCTGATTGCCCGTTTCTTGGCTTGCCTGTGTGCTCTCTTGATAGAGGGGGGCT
GTGAGGGTGATGAAGATCGCATCTCGAG

ClaI –ATCGAT XhoI – CTCGAG yellow – siRNA antisense (390-bp)

AL2 (TrAP) ToMoV – Native

The CDS for the native TrAP overlaps with the REP at the 5', and REN at the 3' with the underlined segment representing the non-overlapping silencing target sequence.

ATGCGATCTTCATCACCCTCACAGCCCCCCTCTATCAAGAGAGCACACAGGCAAGCCAAGAA
ACGGGCAATCAGAAGGCGCAGGGTTGATCTACAGTGCGGGTGCTCCATCTACTTCCACTTAG
ACTGTGCGGGACATGGATTCACGCACAGGGGAACTCATCACTGCACATCAGGCGGAGAATG
GCGTGTATATCTGGGAGCTAGAAAATCCCCTTTATTTCAAGATACACAGAGTAGAGGACCCA
CTGTATACCAGAACGAGGGTATACCACGTACAAATACGGTTCAACCACAACCTGAGGAAAG
CGTTGCATCTCCACAAAGCCTACCTGAACTTCCAAGTTTGGACGACGTCGATGACAGCTTCT
GGATCAATTTATTTAGCTAG

AL2 (cmTrAP) ToMoV – Codon Modified

The codon modification focused on the non-overlap region underlined. Alignments of this region are presented in **Figure 5** of the methods. Red are the modified base pairs:

ATGCGATCTTCATCACCCTCACAGCCCCCCTCTATCAAGAGAGCACACAGGCAAGCCAAGAA
ACGGGCAATCAGAAGGCGCAGGGTTGATTTGCAATGTGGTTGTAGTATTTATTTTCATCTTG
ATTGCGCCGGCCATGGATTCACGCACAGGGGAACTCATCACTGCACATCAGGCGGAGAATG
GCGTGTATATCTGGGAGCTAGAAAATCCCCTTTATTTCAAGATACACAGAGTAGAGGACCCA
CTGTATACCAGAACGAGGGTATACCACGTACAAATACGGTTCAACCACAACCTGAGGAAAG
CGTTGCATCTCCACAAAGCCTACCTGAACTTCCAAGTTTGGACGACGTCGATGACAGCTTCT
GGATCAATTTATTTAGCTAG

NanoLuc Luciferase (Nluc) – Plant Codon Optimized

This luciferase was synthesized based on codon optimization for tomato using the IDT tool, https://sg.idtdna.com/pages/tools/codon-optimization-tool. This is readily available via our ADDGENE vector (Plasmid #212180).

ATGGTGTTTACTCTTGAAGATTTCGTGGGAGACTGGCGTCAAACAGCAGGCTATAACTTAGA
CCAGGTCCTAGAACAGGGAGGCGTTTCCAGTCTGTTTCAGAATCTTGGAGTTAGTGTTACAC
CTATCCAAAGAATAGTTCTCAGCGGTGAAAATGGCCTCAAAATAGACATCCATGTGATCATC
CCATACGAGGGCTTGTCCGGCGATCAAATGGGCCAGATAGAGAAAATATTCAAAGTAGTGT
ACCCAGTAGATGACCACCACTTTAAGGTAATACTTCACTACGGGACCTTAGTTATCGATGGG
GTAACCCCTAATATGATCGACTACTTCGGCCGTCCCTACGAGGGCATTGCTGTCTTCGATGG
GAAGAAGATAACAGTTACCGGCACTTTGTGGAACGGTAACAAAATCATCGATGAGCGACTG
ATCAACCCAGATGGTAGCCTTCTGTTCAGGGTTACCATTAACGGGGTTACAGGGTGGCGTCT
GTGCGAGCGTATATTAGCTTAA

S6: Modeling of Attenuation Vector Feedback Modulation:

Numerical integration of these equations and the associated parameters used in modeling are presented in the DataCommons supplemental Excel spreadsheet.

N_1 – Target deconstructed reporter virus
N_2 – Attenuation vector virus
r_N – virion specific proliferation rate dependence on replication and coat protein
REP - represents the aggregate of replication functions (REP/REN + plant proteins)
CP – represents the coat protein functions
F – feedback parameter (silencing RNA)

Virus Proliferation – Baseline Model

This is a simple proliferation model for the virus based on a maximum capacity (K=2000 virions / cell). To achieve a steady state, all terms must have a degradation term (βi) where rate of protein degradation (β_{pr}) is treated differently from the degradation of other model elements.

$$\frac{dN_1}{dt} = r_N\left[N_1\left(1 - {N_1}/{K}\right)\right] - \beta_V N_1$$

$$r_N = r_{max}\left(\frac{REP}{k_{rep} - REP}\right)\left(\frac{CP}{k_{cp} - CP}\right)$$

$$\frac{d[REP]}{dt} = \alpha_{rep}N_1 - \beta_{pr}[REP]$$

$$\frac{d[CP]}{dt} = \alpha_{cp}N_1 - \beta_{pr}[CP]$$

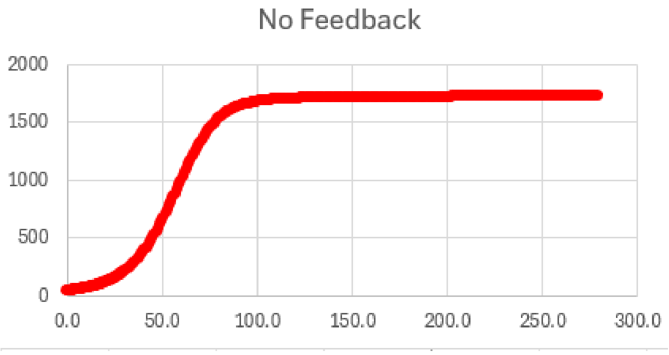

Virus Proliferation – Attenuation Vector (N_1 Feedback)

In this model a generalized feedback repression is introduced which is specifically focused on the deconstructed virus (N_1) with the feedback element (F) being produced by the replacement silencing element driven by the coat protein promoter (α_{cp}) in the attenuation vector (N_2). The capacity for virion production is shared between the two viral forms ($K-N_j$).

$$\frac{dN_1}{dt} = r_N\left[N_1\left(1 - {N_1}/{(K - N_2)}\right)\right] - \alpha_f[F]N_1 - \beta_V N_1$$

$$\frac{dN_2}{dt} = r_N\left[N_2\left(1 - {N_2}/{(K - N_1)}\right)\right] - \beta_V N_2$$

$$r_N = r_{max}\left(\frac{REP}{k_{rep} - REP}\right)\left(\frac{CP}{k_{cp} - CP}\right)$$

$$\frac{d[REP]}{dt} = \alpha_{rep}(N_1 + N_2) - \beta_{pr}[REP]$$

$$\frac{d[CP]}{dt} = \alpha_{cp}N_1 - \beta_{pr}[CP]$$

$$\frac{d[F]}{dt} = \alpha_{cp}N_2 - \beta_f[F]$$

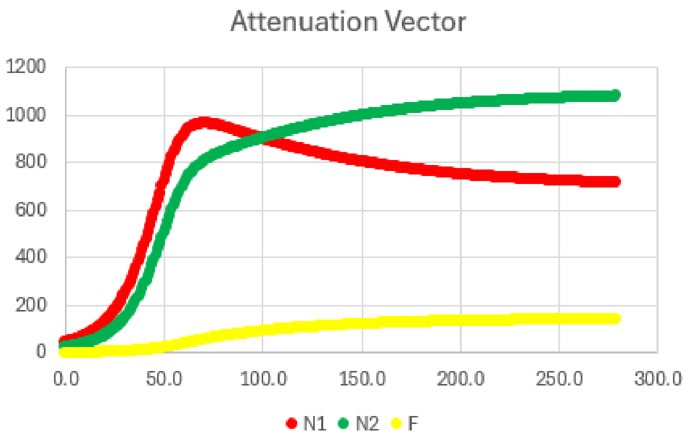

Virus Proliferation – Attenuation Vector (REP silencing)

In this model the feedback repression is focused on the silencing of the replication functions (and assumes the TrAP is adequately provided by the attenuation vector (N_2) The capacity for virion production is shared between the two viral forms as in the previous model.

$$\frac{dN_1}{dt} = r_N\left[N_1\left(1 - {N_1}/{(K - N_2)}\right)\right] - \beta_V N_1$$

$$\frac{dN_2}{dt} = r_N\left[N_2\left(1 - {N_2}/{(K - N_1)}\right)\right] - \beta_V N_2$$

$$r_N = r_{max}\left(\frac{REP}{k_{rep} - REP}\right)\left(\frac{CP}{k_{cp} - CP}\right)$$

$$\frac{d[REP]}{dt} = (\alpha_{rep} - \alpha_F[F])N_1 + \alpha_{rep}N_2 - \beta_{pr}[REP]$$

$$\frac{d[CP]}{dt} = \alpha_{cp}N_1 - \beta_{pr}[CP]$$

$$\frac{d[F]}{dt} = \alpha_{cp}N_2 - \beta_f[F]$$

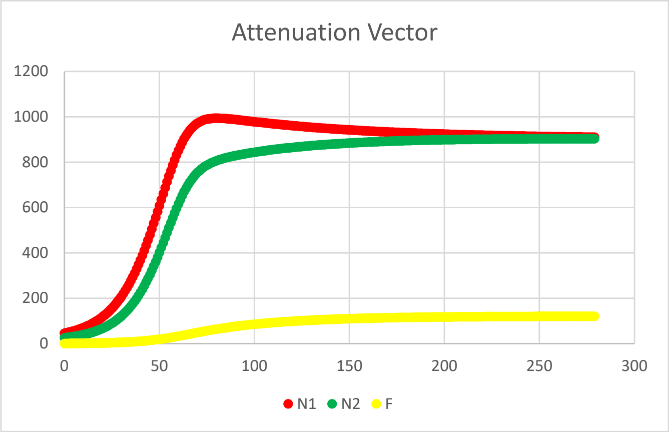

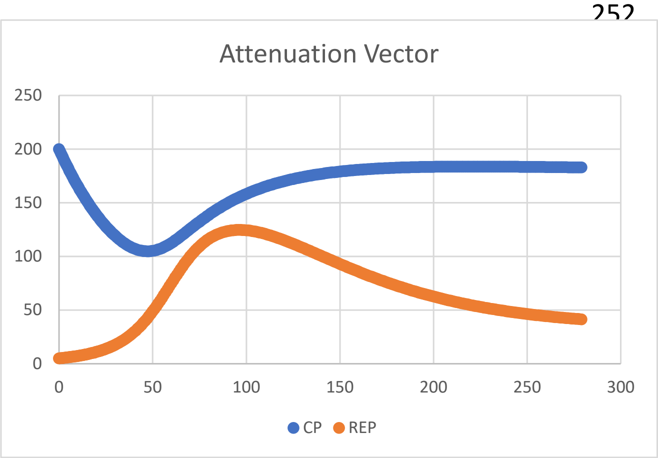

S7: Silencing of eGFP Viral vector – Original Southern Blot for Figure 3e:

As described in Figure 3, virus silencing was tested via simple expression of siRNA constructs constitutively from a binary vector using an available eGFP+ coat protein replacement A-component of closely related pepper golden mosaic virus (PepGMV). The original Southern blot on nitrocellulose membrane is provided below. Fragments homologous to AL1-AL3 (nt1696 to 1202) were introduced by agrobacterium infiltration for silencing. For the single copy (pFGC1008-ToMoVALforward), expression from the 35S promoter produces a virion sense small RNA to anneal with complementary sense virion mRNA encoding AL1, AL2 and AL3. For the inverted repeat (pFGC1008-dsToAL), expression from the 35S promoter will produce an RNA capable of forming a stem-hairpin substrate for DICER and RISC to generate the siRNA.

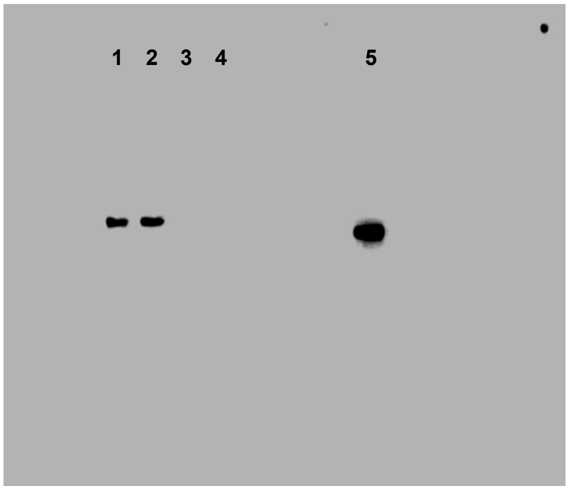

Lane **1**: eGPF Viral Vector (pTPVA1.5gfp)
Lane **2**: pTPVA1.5gfp + empty silencing vector (pFGC1008)
Lane **3**: pTPVA1.5gfp + silencing (pFGC1008-PePALfor)
Lane **4**: pTPVA1.5gfp + silencing (pFGC1008-dsPePAL)
Lane **5**: positive control (YFP PCR Product)

Figure S7: Testing of the ability of reporter viral vector PePGMV-1.5 CP-/eGFP+ to replicate and accumulate viral DNA in presence and absence of complementary strand gene silencing. Total DNA isolated 3-days post-infiltration from Agro-infused *N. benthamiana* leaves was run on an agarose gel and probed with GFP chemiluminescence probe (Southern blot). Original Southern blot on nitrocellulose membrane from LiCor C-Digit scanner (signal so strong that there is no contrast for membrane edges other than spot on upper right corner). Replicating viral DNA was detected in the two control samples lacking the silencing vectors (Lanes 1 and 2). Viral DNA was below the levels of detection in the two samples containing the either the simple siRNA or the hairpin siRNA constructs (Lanes 3 and 4 respectively). YFP generated as a PCR product was run as a positive control (Lane 5).
